# Immunoinformatics and computational approaches driven designing a novel vaccine candidate against Powassan virus

**DOI:** 10.1038/s41598-024-56554-9

**Published:** 2024-03-12

**Authors:** Truc Ly Nguyen, Heebal Kim

**Affiliations:** 1 Department of Agricultural Biotechnology and Research Institute of Agriculture and Life Sciences, Seoul National University, Seoul, 08826 Republic of Korea; 2 Interdisciplinary Program in Bioinformatics, Seoul National University, Seoul, 08826 Republic of Korea; 3 eGnome, Inc., Seoul, 05836 Republic of Korea

**Keywords:** Powassan virus, Arboviruses, Multi-epitope vaccine, Immunoinformatics, Zoonotic diseases, Computational vaccinology, Peptide vaccines, Protein design, Protein structure predictions, Viral infection

## Abstract

Powassan virus (POWV) is an arthropod-borne virus (arbovirus) capable of causing severe illness in humans for severe neurological complications, and its incidence has been on the rise in recent years due to climate change, posing a growing public health concern. Currently, no vaccines to prevent or medicines to treat POWV disease, emphasizing the urgent need for effective countermeasures. In this study, we utilize bioinformatics approaches to target proteins of POWV, including the capsid, envelope, and membrane proteins, to predict diverse B-cell and T-cell epitopes. These epitopes underwent screening for critical properties such as antigenicity, allergenicity, toxicity, and cytokine induction potential. Eight selected epitopes were then conjugated with adjuvants using various linkers, resulting in designing of a potentially stable and immunogenic vaccine candidate against POWV. Moreover, molecular docking, molecular dynamics simulations, and immune simulations revealed a stable interaction pattern with the immune receptor, suggesting the vaccine's potential to induce robust immune responses. In conclusion, our study provided a set of derived epitopes from POWV’s proteins, demonstrating the potential for a novel vaccine candidate against POWV. Further in vitro and in vivo studies are warranted to advance our efforts and move closer to the goal of combatting POWV and related arbovirus infections.

## Introduction

Emerging infectious diseases have posed significant challenges to public health worldwide in recent years. Among these, the POWV is a compelling subject of investigation due to its potential to cause severe neurological illness in humans. POWV, a member of the Flaviviridae family, belongs to a group of vector-borne viruses that includes other lethal pathogens such as Zika, dengue, and West Nile virus^1^. The Powassan genome is composed of about 11 kb of single-stranded, positive-sense RNA that encodes three structural proteins: the capsid (C) protein, the premembrane (prM) protein, and the envelope (E) glycoprotein at the 5′ end of the genome and seven nonstructural proteins in the 3' end of the genome^2^. Geographically, the virus is predominantly found in North America, with a higher incidence in the eastern parts of Canada, Northeastern and Great Lakes regions of the United States, and in far eastern Russia^2,3^. There are two lineages of POWV: lineage 1, which includes POWV strain LB, and lineage 2, also called deer tick virus^4^. The lineage 1 virus is primarily transmitted to humans through the bite of infected ticks, specifically the *Ixodes scapularis* tick (also known as the black-legged tick) in North America. Meanwhile, the lineage 2 virus, such as *Ixodes cookei* mostly transmits to humans in the far eastern region of Russia^5^.

The virus can lead to various clinical presentations, from asymptomatic cases to severe neuroinvasive disease^6^. The time between being bitten by an infected tick and the onset of symptoms (incubation period) can vary, but it's generally within 1–5 weeks^2,3^. Many individuals who are infected with POWV may not show any symptoms. Those who develop symptoms can range from mild to severe, including fever, headache, vomiting, weakness, and confusion. In some cases, neurological symptoms such as encephalitis (inflammation of the brain) and meningitis (inflammation of the membranes surrounding the brain and spinal cord) can occur^7^. Given the potentially rapid onset of symptoms and the lack of specific antiviral therapies, understanding the factors contributing to disease severity and developing novel strategies to prevent and treat POWV is paramount. Preventing POWV infections hinges on effective tick bite prevention strategies, including insect repellents, protective clothing, and thorough tick checks^8^. As for treatment, there is no specific antiviral therapy or vaccine for Powassan virus infections. Supportive care is the cornerstone of managing cases, and hospitalization may be required for severe presentations^9^.

In response to the urgent need for effective countermeasures against POWV infections, this study takes an innovative immunoinformatics approach to design a potential vaccine candidate. Leveraging the power of computational tools and bioinformatics analyses, we aim to harness the immune system's capabilities by targeting the structural proteins of POWV. These proteins, including the capsid, envelope, and membrane proteins, play pivotal roles in virus entry, replication, and host interactions, making them attractive targets for vaccine design. Initial steps encompass the retrieval and analysis of structural protein sequences of POWV to identify B-cell epitopes and major histocompatibility complex class I (MHC-I) and MHC-II binding peptides. By employing algorithms that predict binding affinity to MHC molecules, we prioritize epitopes likely to be presented to T cells, a key step in generating cellular immunity. Then, the antigenicity, allergic potential, toxicity, and cytokine inducing potential of the epitopes were predicted to ensure that our vaccine candidate triggers specific immune responses without causing adverse effects. We aim to narrow the list of potential epitopes exhibiting optimal immunological properties through these stringent screening processes. Subsequently, we design a vaccine candidate using the epitopes that satisfy our selection criterion by joining them to adjuvants using suitable linkers. Finally, we employ molecular modeling techniques to generate the vaccine candidate's three-dimensional structure, which is then used to perform molecular docking and molecular dynamics simulation analyses with toll-like receptor (TLR) molecules.

## Results

### Retrieval and analysis of protein sequences

The 3415 amino acid long polyprotein sequence reviewed has protein level evidence and an annotation score of 5/5. In the polyprotein sequence, the capsid protein ranges between 1 and 94 amino acids, prM/M protein ranges between 116 and 278 amino acids, and envelope protein ranges between 279 and 775 amino acids. All the structural proteins were predicted as antigenic by the Vaxijen 2.0 tool. Similarly, all the structural protein except capsid protein was determined as non-allergic by the AllergenFP webserver.

### Prediction and evaluation of B-cell and T-cell epitopes

A total of 67 strong binding MHC-I epitopes were identified, including 12 epitopes from capsid protein, 17 from prM/M protein, and 38 from envelope protein. Similarly, 40 strong binding MHC-II epitopes were predicted from the capsid protein, 14 from the prM/M protein, and 52 from the envelope protein, taking the total of strong binding MHC-II epitopes to 106. Finally, from the structural protein, 19 B-cell epitopes were predicted. All these epitopes were then evaluated for their antigenicity, IL-4, and Interferon-gamma inducing ability, toxicity, and allergenicity (Supplementary Datasets). After all the analysis, 8 epitopes were predicted: 2 from MHC-I, 4 from MHC-II, and 2 from B-cell epitopes that were antigenic, non-toxic, non-allergic, and could activate IL-4 and Interferon-gamma (Table 1).

**Table 1:** Final epitopes selected for the vaccine design.

Type of epitope	Protein	Epitope
MHC-I	Envelope	DTVVMEVSY
		VEFGPPHAV
MHC-II	Capsid	MFWKTVPLRQAEAVL
		MFWKTVPLRQAESAL
	Envelope	GSTIGRMFEKTRRGL
		GSTIGRMFEKTRKGL
B-cell	Envelope	KHKDNQDWNS
		EFGPPHAV

### Formulation of the vaccine candidate and determination of the properties

**Figure 1:**
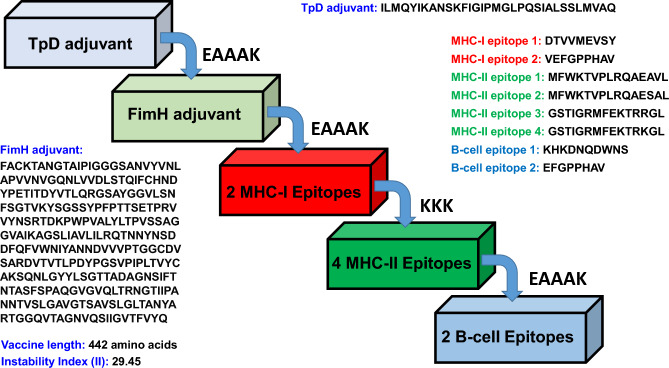
Schematic representation of the vaccine design with linkers, adjuvant, and epitopes sequentially and appropriately.

Eight chosen epitopes highly suitable for vaccine design were connected with adjuvants via stable linkers. As a result, the final vaccine candidate has 442 amino acids (Fig. 1). Furthermore, the vaccine candidate was predicted as antigenic by the Vaxijen 2.0 webserver and non-allergic by the AllergenFP webserver. The negative GRAVY score indicates that the vaccine candidate is hydrophilic, and the instability index of 29.45 below 40 shows that the vaccine candidate is stable (Table 2). Also, the final vaccine construct has no transmembrane helices or signal peptides. Remarkably, in the BLASTp analysis, the vaccine candidate appears distinct from human proteins, suggesting that it has the potential to be used safely in humans without triggering an autoimmune response in the host.

**Table 2:** Physiochemical properties of the formulated vaccine candidate.

Features	Assessment	Remark
Molecular weight	47,250.12	Average
Number of amino acids	442	–
Signal peptide	0	–
Transmembrane helix	0	–
Total number of atoms	6685	–
Extinction coefficient	54,570	Average
Theoretical pI	9.65	Slightly basic
Aliphatic index (AI)	82.29	Thermostable
Instability Index (II)	29.45	Stable
Estimated half-life (mammalian reticulocytes, in vitro)	20 h	Satisfactory
Estimated half-life (yeast cells, in vivo)	30 min	Satisfactory
estimated half-life (*Escherichia coli*, in vivo)	> 10 h	Satisfactory
Grand Average of hydropathicity (GRAVY)	− 0.099	Hydrophilic
Antigenicity	0.6346 (VaxiJen v2.0)	Antigenic
Allergenicity	Non-allergen (AllergenFP)	Non-allergen

### In-silico immune simulation of the vaccine candidate

**Figure 2:**
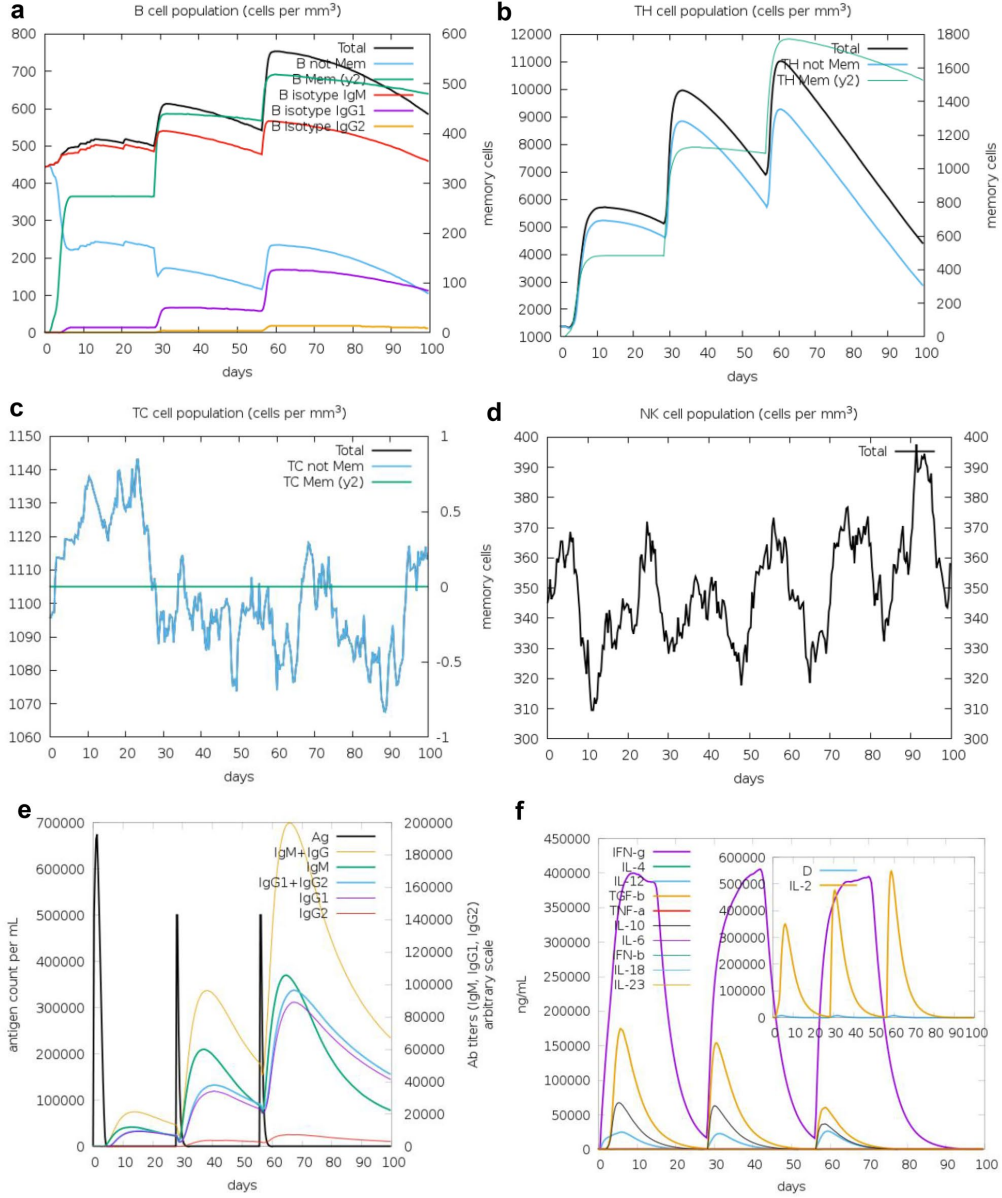
Powassan virus vaccine immune-simulation. (**a**) B-cell population. (**b**) T-helper cell population. (**c**) T-cytotoxic cell population. (**d**) Nature killer cell population. (**e**) Antigen count and antibody titer with specific subclass. (**f**) Concentration of cytokines and interleukins. Inset plot shows danger signal together with leukocyte growth factor IL-2.

Figure 2 visually represents the anticipated immune response pattern resulting from the formulated vaccine, as determined through computational analysis. Successive administrations of the vaccine trigger an expansion in both the total B-cell population and B-memory cell populations, highlighting the stimulation of a robust secondary immune response (Fig. 2a). Following each vaccination the total T-helper (TH) cell population and T-helper memory cells increased (Fig. 2b). On the other hand, the T-cytotoxic (TC) memory cell population rises after the first vaccination but notably decreases after the second and third doses (Fig. 2c). Besides, the proliferation of natural killers (NK) was also observed, which is essential mediators of T cells activation (Fig. 2d). Furthermore when examining the impact of the initial dose (Fig. 2e) in comparison to the subsequent second and third doses, it becomes evident that there is an increase in the concentrations of various antibodies, including IgM + IgG, IgM, IgG1 + IgG2, IgG1, and IgG2, indicating that immunization with the candidate vaccine leads to an augmented antibody response. Additionally, the Powassan vaccination using the formulated vaccine candidate can induce the generation of various cytokines, including IFN-gamma, interleukin-10 (IL10), interleukin-12 (IL12), and transforming growth factor-beta (TGF-β) (Fig. 2f). In comparison to the initial dose, the second dose of the vaccine results in increased populations of IFN-gamma, IL-10 and IL-12 but showed a decrease in the population of TNF-alpha. After receiving the third dose of the vaccine construct, there is an overall decrease in the concentration of different cytokines and interleukins compared to the first and second doses.

### Modeling, refinement, and validation tertiary structure of the vaccine candidate

The vaccine construct was modeled using the cutting-edge AlphaFold program^10,11^. Given that the AlphaFold prediction yielded a local distance difference test score (pLDDT) below 70 (Fig. S1) and the Ramachandran plot analysis revealed only 79.8% of residues in most favoured regions (Fig. S2), we conducted further structural refinement employing the GalaxyRefine server^12^. Refer to Table 3 for the refined model's assessment, Model 2 demonstrated the highest global distance test-high accuracy (GDT-HA) score of 0.9632 (the higher value, the more accurate), and the lowest root mean square deviation (RMSD) score of 0.397 (lower value indicating greater stability).

**Table 3:** Structure Information obtained from GalaxyWEB and ERRAT program.

Model	GDT-HA	RMSD	MolProbity	Clash score	Poor rotamers	Rama favored	ERRAT value
Model 1	0.9581	0.448	1.518	8.5	0.0	97.7	94.0789
Model 2	0.9632	0.397	1.498	8.0	0.3	97.7	95.7790
Model 3	0.9587	0.444	1.524	8.6	0.0	97.7	92.0630
Model 4	0.9570	0.460	1.488	8.9	0.6	98.0	92.6984
Model 5	0.9570	0.477	1.531	8.8	0.3	97.7	94.1558

**Figure 3:**
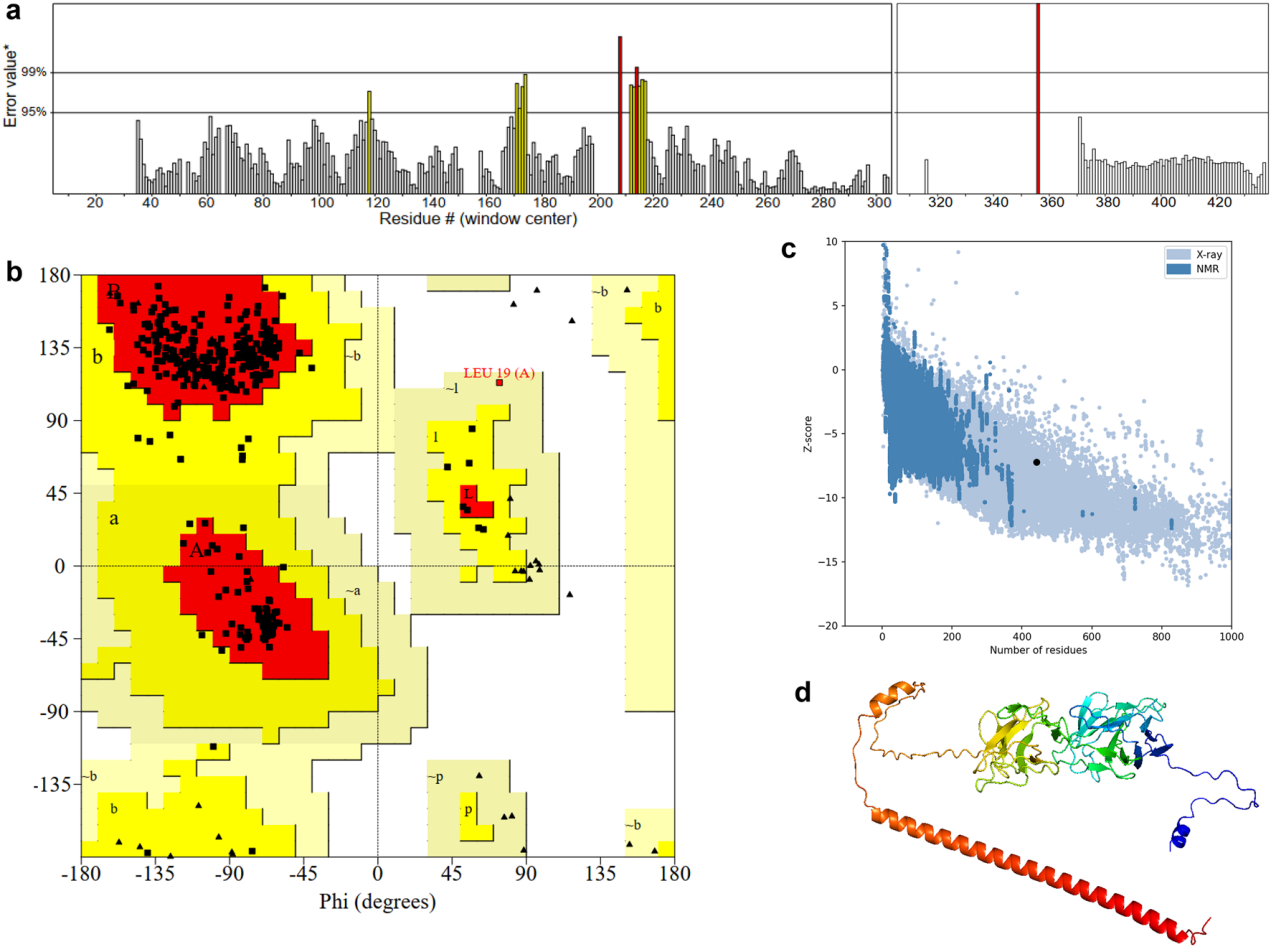
Tertiary structure of the vaccine candidate. (**a**) ERRAT program plot. (**b**) Ramachandran plot by PROCHECK webserver. (**c**) Z-score calculated by Pro-SA webserver. (**d**) Tertiary structure of refined Model 2 refined by GalaxyRefine, colored in rainbow from N-terminal (in blue) to C-terminal (in red).

Besides, among the 5 models, only Model 2 had an ERRAT value greater than 95% (Table 3 and Fig. 3a). According to ERRAT program, good high resolution structures generally produce values around 95% or higher. Furthermore, to validate the model's quality, we analyzed the Ramachandran plot and calculated a Z-score. The Ramachandran plot analysis revealed that 94.7% of residues were situated in the favorable core region, with 5.3% in the allowed and generously allowed regions, and 0.0% in the disallowed region (Fig. 3b). Meanwhile, the Z-score had a value of -7.02 using the ProSA webserver (Fig. 3c). Hence, we can infer that the tertiary structure of refined Model 2 (Fig. 3d) exhibits a high level of quality suitable for subsequent docking studies.

### Molecular docking of the vaccine candidate and immune receptor

**Figure 4:**
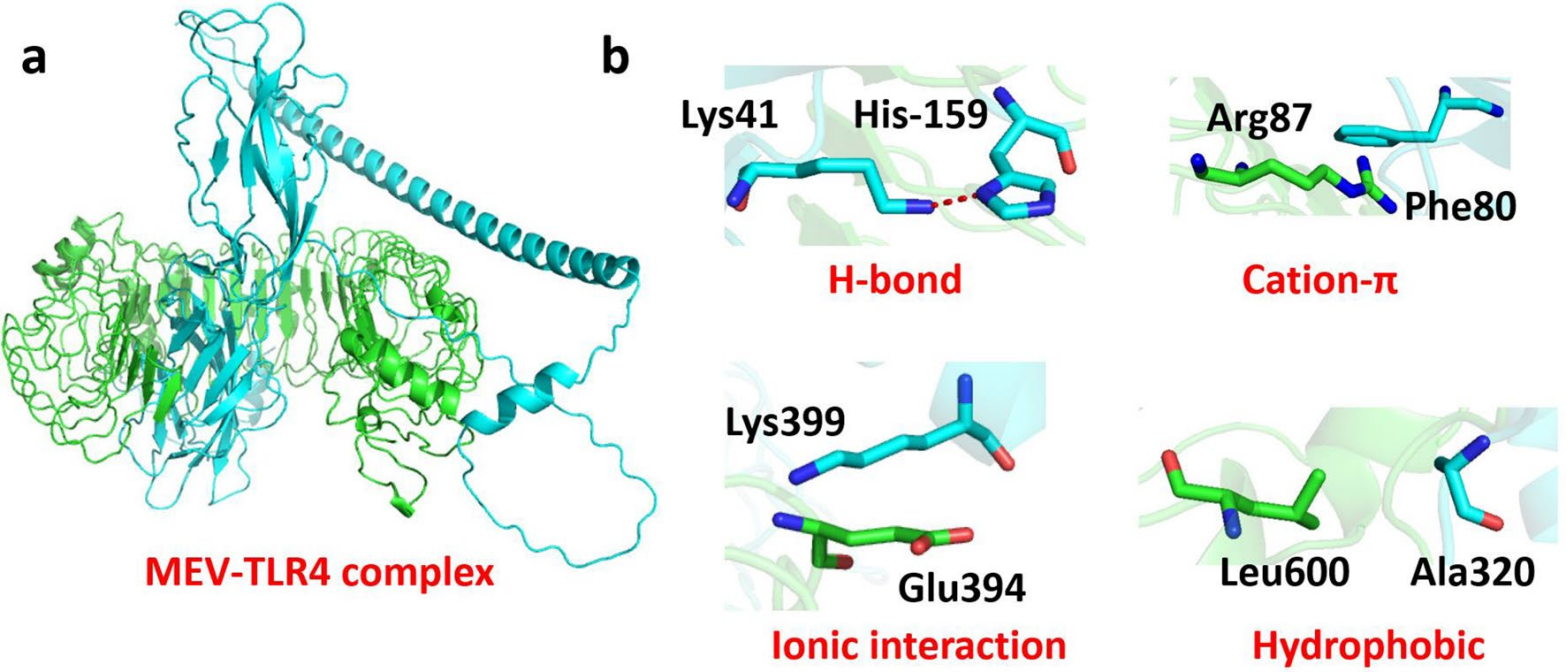
(**a**) Molecular docking between Powassan vaccine and TLR4 with docked complex. (**b**) Four distinct inter-molecular representative interactions between vaccine and TLR4 complex are in sticks representation.

Moving forward, we acquired the tertiary structure for the human TLR4 receptor (Uniprot ID: O00206) from the AlphaFold database. In the case of the TLR4 structure, we retained only the extracellular domain encompassing amino acids 30–624, excluding other regions. For the docking of vaccine with TLR4, we utilized the PatchDock webserver, adhering to default settings^13^. The docked structure is illustrated in Fig. 4, with four representative interactions highlighted in stick representation formed between the vaccine and TLR4 complex.

**Figure 5:**
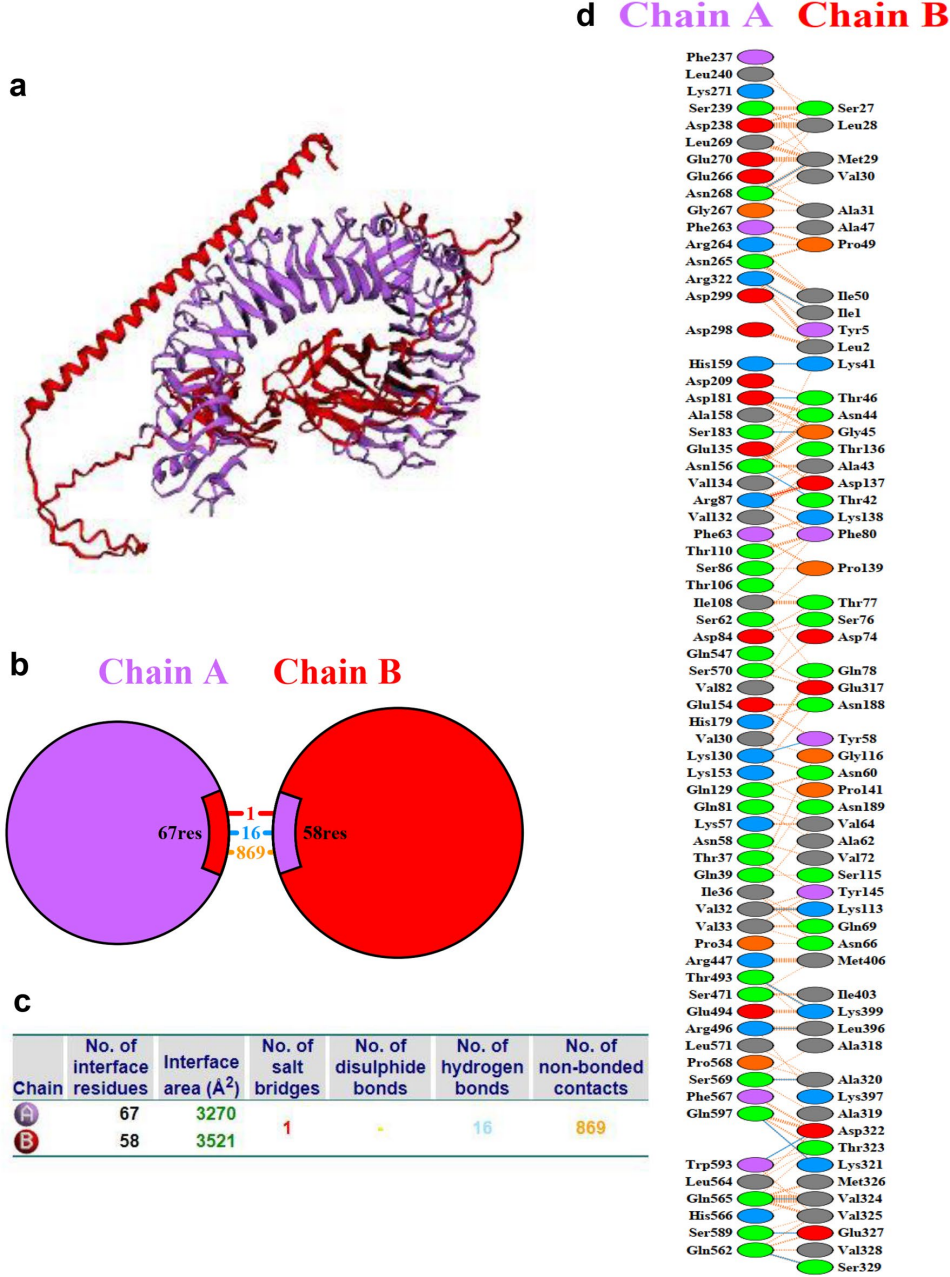
Molecular docking between TLR4 (purple) and vaccine (red). (**a**) Three-dimensional representation of molecular docking. (**b**) Schematic diagram of interactions between protein chains. (**c**) Interface statistics. (**d**) Residue interactions between TLR4 (chain A) and vaccine (chain B), salt-bridge (red line), hydrogen bonds (blue lines), and non-bonded contacts (orange dashed lines) between residues on either side of the vaccine-receptor interface.

Besides, interacting residues between the vaccine and TLR4 were analyzed using the PDBsum web server^14^. There are 67 and 58 residues interface, generating interface area 3270 and 3521 (Å) between TLR4 and vaccine, respectively. As a result, a total of 16 hydrogen bonds (blue lines), 1 salt bridge (red line), and 869 non-bonded contacts (orange dashed lines) were indicated in Fig. 5 and Table S.

### Molecular dynamics simulations

**Figure 6:**
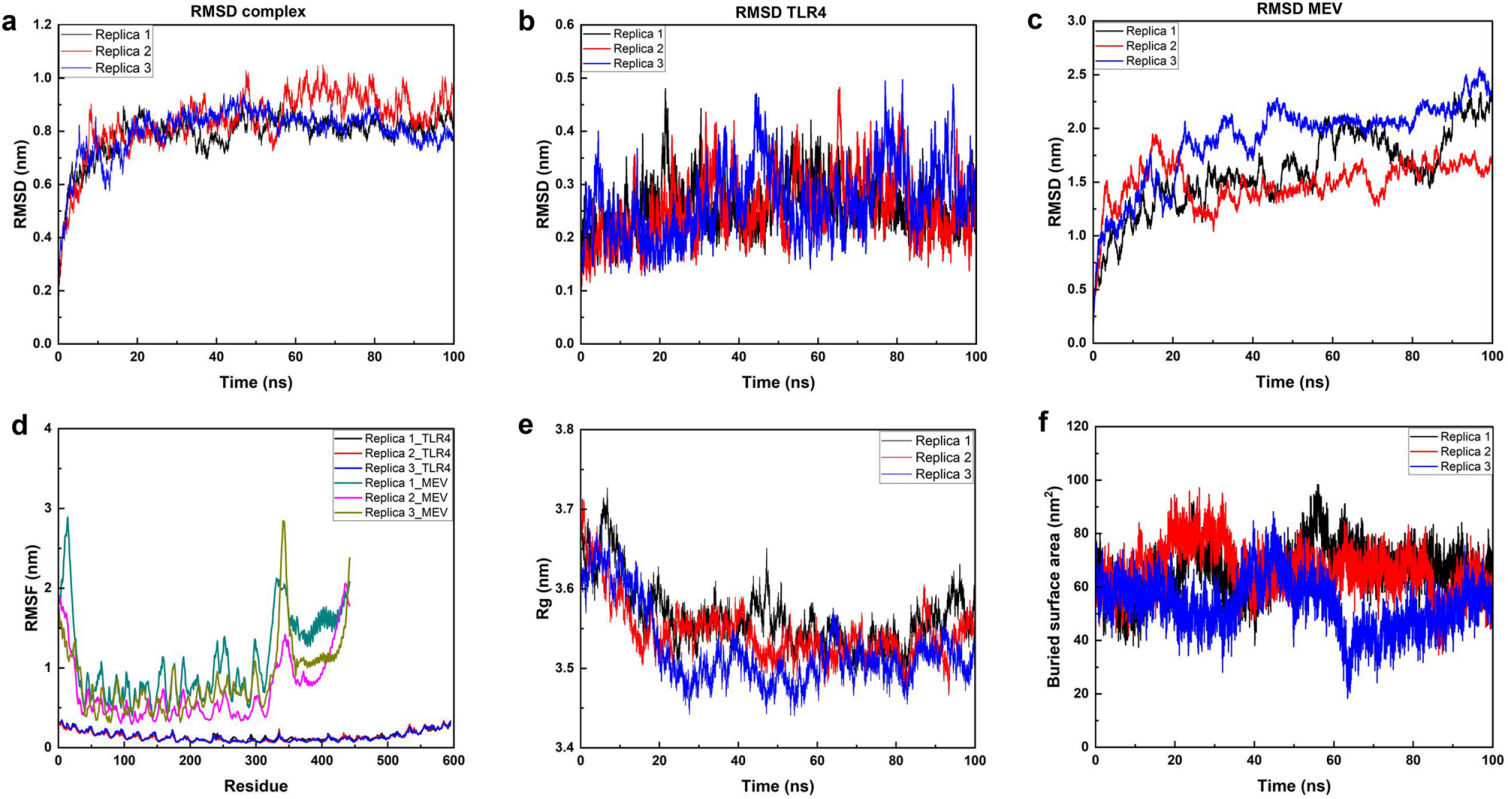
Molecular dynamics simulation studies. (**a**–**c**) RMSD backbone of the docking complex, TLR4, and MEV, respectively. (**d**) RMSF of the docking complex. (**e**) Rg in total of the docking complex. (**f**) BSA of the docking complex.

To assess the stability of interactions in the vaccine and TLR4 complex, three replicas with different initial velocities throughout 100 ns of the MD simulations were run for all-atom using GROMACS 2023 software. Highly conserved results were achieved in all three replicates, as shown in Fig. 6. For clarity, we will only report the results for the first replication. The root mean square deviation (RMSD) from the backbone of the complex, TLR4, and MEV were analyzed with average values of 0.786 ± 0.087 nm, 0.259 ± 0.046 nm, and 1.599 ± 0.375 nm respectively (Fig. 6a–c). Besides, the root mean square fluctuation (RMSF) of the complexes was quantified to recognize the flexibility across the amino acid residues from the complexes. As depicted in Fig. 6d, the RMSF values for TLR4 and MEV were calculated at 0.143 ± 0.057 nm and 1.109 ± 0.518 nm, respectively, indicating higher flexibility in the vaccine than TLR4. Subsequently, the radius of gyration in total (Rg) from the simulation complex was also analyzed to examine the mobility and overall flexibility of the complex. Figure 6e shows that the complex had lower aberrations from 0 to 20 ns, and then the complex was stabilized and maintained the firm profile with a value of 3.565 ± 0.041 nm. Furthermore, the buried surface area (BSA) of the vaccine and TLR4 complex was calculated. The BSA for each residue of the docking complex was averaged over 100 ns to ensure the system has reached an energy-minimized state. Throughout the simulation, the BSA at the interaction interface between the MEV and TLR4 remained stable in all three repetitions with an average value of 66.460 ± 8.982 nm^2^ (Fig. 6f).

**Figure 7:**
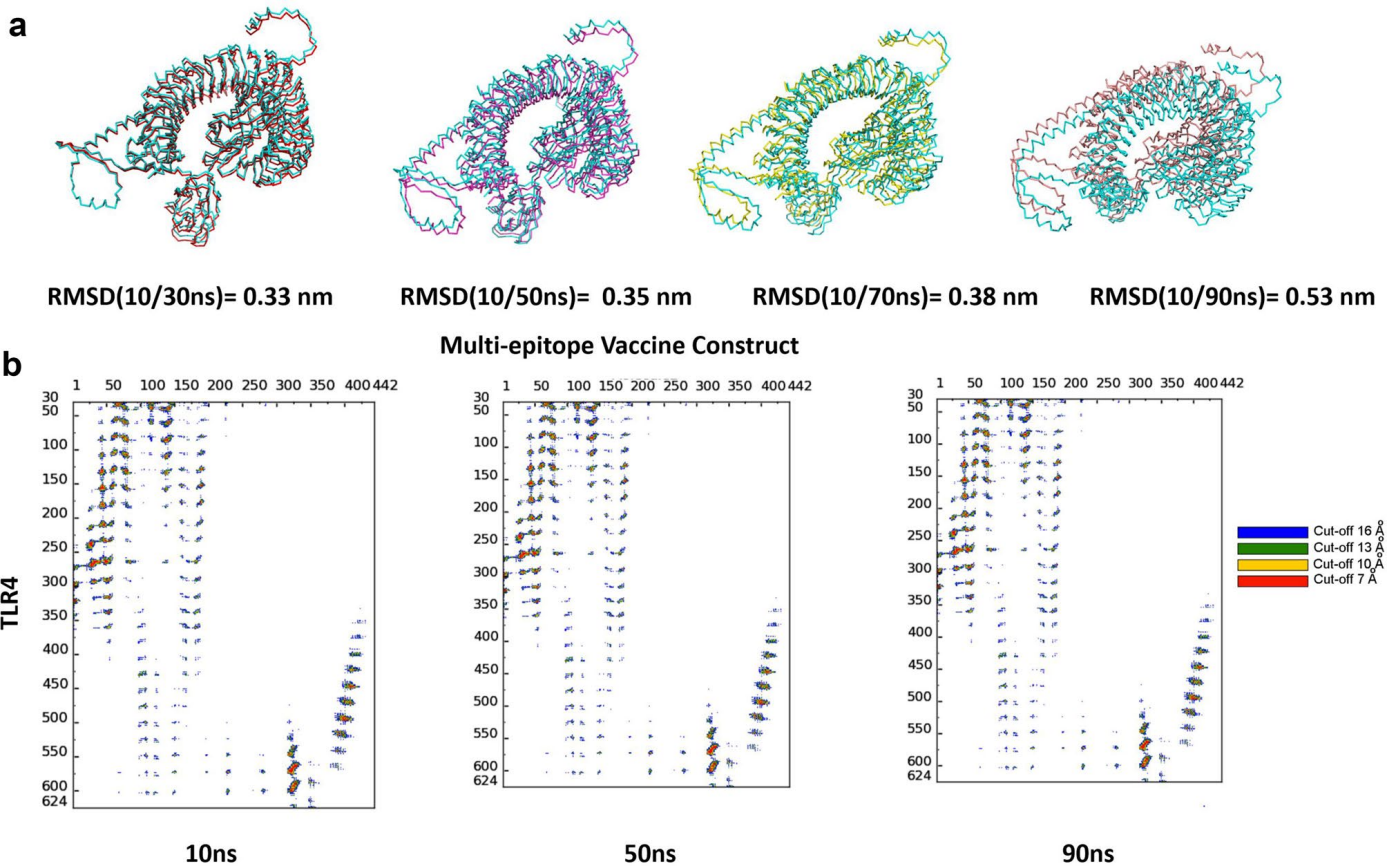
(**a**) Overlaying specific snapshots of the TLR4 and vaccine construct, along with their corresponding RMSD values in the initial simulation run. (**b**) Contact maps visually represent intermolecular interactions, indicating proximity between atom pairs within defined distances. Dots at the junction of two residues are color-coded, with red, yellow, green, and blue denoting closeness within 7, 10, 13, and 16 Å, respectively.

Additionally, the structural integrity's robustness was confirmed by aligning the entire complex involving the vaccine construct and the TLR4 receptor. Figure 7 illustrates a favorable alignment between these structures. The observed higher RMSD values in Fig. 7a are attributed to the presence of unaligned, flexible regions, particularly the long and flexible loops within the vaccine construct. Despite these structural variations, the interaction pattern between the vaccine and TLR4 remains consistently stable. Besides, to further explore the interface connecting TLR4 and the vaccine construct, like other researchers^15,16^, specific snapshots were analyzed using the COCOMAPS tool as shown in Fig. 7b. This analysis produced contact maps, visually representing the pairwise distances between residues of the vaccine construct and TLR4. In these contact maps, dots are color-coded: red, yellow, green, and blue signify distances below 7, 10, 13, and 16 Å, respectively.

## Discussion

POWV was discovered for the first time in Powassan, Canada, by an encephalitis patient in 1958^17^. Usually, it causes sporadic infections, but since 2007, its cases have increased steadily in North America^3^. The frequency of POWV incidents stood at 0.7 per year between 1958 and 1998, increased to 1.3 cases annually spanning 1999 to 2005, and surged to 7.7 cases per year from 2006 to 2015^18^. Moreover, the incidence rate of POWV has increased by 300% in the previous two decades^19^. Unlike some other tick-borne diseases, POWV can be transmitted within 15 min after the bite of an infected tick^6^. It leads to persistent neurological consequences in approximately 50% of documented cases and results in fatality in slightly over 10% of the cases^18^.

There are already six licensed vaccines for the flavivirus tick-borne encephalitis virus that can prevent the onset of neurological sequelae and severe disease; however, these vaccines are ineffective against POWV infection^5^. No medicine or vaccine is currently approved for treating and preventing POWV disease. However, researchers continuously explore novel avenues to develop vaccine candidates or therapies for Powassan virus treatment. Antiviral molecules against flaviviruses, namely the adenosine analogue NITD008 and NS5 capping enzyme inhibitor BG323, exhibited noteworthy reductions in POWV levels in vitro studies^20,21^. Intravenous immunoglobulin therapy has been utilized to manage POWV encephalitis for two patients. In both cases, the administration of intravenous immunoglobulin led to the survival of the patients with POWV infection. However, it's worth noting that one of the patients experienced considerable neurological complications following their recovery and discharge^22^. Recently, a vaccine candidate that uses the yellow fever virus vaccine strain as a vector expresses the prM/M and envelope proteins of POWV protected mice following lethal challenge and conferred a survival rate of 70%^23^.

Similarly, in another study, an mRNA vaccine encoding PrM/M and envelope proteins of POWV encapsulated in lipid nanoparticles generated neutralizing antibodies and protected mice from the lethal challenge of POWV^24^. In another study, Choi et al. developed a synthetic DNA vaccine consisting of POWV prM and envelope proteins, which elicited T cell and B cell immunity in mice and also protected the mice from POWV lethal challenge^19^. Additionally, an immunogen displaying the domain III of the envelope glycoprotein of POWV presented on self-assembling protein nanoparticle induced protective and neutralizing antibodies against POWV in mice^25^.

Developing a vaccine against POWV is crucial due to the potential severity of its neurological infections, including encephalitis and meningitis, often leading to long-term health consequences or even death. As an emerging tick-borne virus with increasing incidence over the years, the absence of specific antiviral treatments highlights the urgent need for preventive measures. A vaccine candidate against the POWV would provide a proactive solution for safeguarding public health and reducing healthcare burdens, aiding in curtailing the virus's geographical expansion and protecting vulnerable populations. Hence, in this study, a vaccine candidate has been developed using immunoinformatics approaches by targeting the structural proteins of POWV. Previously, several studies have targeted the Powassan structural proteins, mainly prM/M and envelope proteins, for developing vaccine candidates using strategies^19,23,24^. Lately, there has been a notable rise in the adoption of immunoinformatics in creating vaccine candidates. This computational methodology offers a time- and cost-efficient means of developing innovative vaccine candidates. Another benefit of this method is its ability to identify numerous potential vaccine candidates without the need for cultivating pathogenic organisms in a traditional laboratory setting^26,27^.

Moreover, comparable immunoinformatics techniques have been applied to formulate vaccine candidates targeting Monkeypox, Canine circovirus, Human cytomegalovirus, and Dengue virus^28–31^. Previously, one study has used the immunoinformatics approach to identify B-cell and T-cell epitopes only and did not evaluate the properties such as antigenicity, allergic potential, cytokine cytokine-inducing potential^32^. Furthermore, the epitopes predicted in our study and the previous study were not similar, and the previous research did not design the final vaccine construct. The final epitopes selected in our study are antigenic, non-allergic, non-toxic and can induce various cytokine generation. Furthermore, the vaccine candidate formulated could be effective in generating an immune response in both the POWV lineages as both MHC-I epitopes and B-cell epitopes used in the final vaccine design are present in both lineage I and lineage II of the POWV. Furthermore, the two MHC-II epitopes from POWV lineage II that have high sequence similarity with the final two MHC-II epitopes used in the vaccine construct have been added to the formulated vaccine construct to make the vaccine effective against lineage II virus as well.

To enhance the efficacy of the final vaccine construct, the final subunit of the *Escherichia coli* type 1 fimbria (FimH) and epitope of tetanus and diphtheria toxoid (TpD) adjuvants were used. Laboratory experiments have shown that TpD can stimulate the production of neutralizing antibodies and protect mucous membranes^33,34^. *E. coli* type 1 FimH has been reported to interact with TLR4, elicit the generation of IFN-γ and TNF-α, and play a role in the proliferation of local dendritic cells^35,36^. Finally, the formulated POWV vaccine candidate was determined as antigenicity, toxicity, and allergenicity. Proteins with a molecular weight below 110 kDa are regarded as appropriate vaccine candidates^26^. The formulated vaccine candidate had a molecular weight of 47.2 kDa, confirming its suitability as a vaccine candidate. Moreover, the vaccine candidate does not have homology with any human protein, thus minimizing the risk of autoimmune response to the hosts. The final vaccine protein exhibited an instability index of 29.45, suggesting its stability in biological conditions, as compounds with an instability index < 40 are considered stable. Additionally, according to computational immune simulation, the formulated POWV vaccine candidate is expected to have the potential to trigger strong immune responses in those who receive it.

To delve deeper into the relationship between the vaccine candidate and TLR4, we conducted molecular docking and molecular dynamics simulations which verified the stability of these interactions. Three-dimensional representation of molecular docking between the vaccine candidate and TLR4 is shown in Fig. 5a. Interestingly, the area of each circle is proportional to the surface area of the corresponding protein chain. The extent of the interface region on each chain is represented by the black wedge whose size signifies the interface surface area (Fig. 5b). Statistics for this interface are given in Fig. 5c with numbers of interface residues 67 (TLR4) and 58 (MEV), interface area of 3,270 (Å^2^) and 3,521 (Å^2^). Notably, interacting chains between two structures are joined by colored lines, each representing a different type of interaction, as per the key: salt-bridge (red line), hydrogen bonds (blue lines), and non-bonded contacts (orange dashed lines) (Fig. 5d).

Furthermore, the root mean square deviation (RMSD) of the backbone and root mean square fluctuations (RMSF) of the C-alpha atoms for each residue were calculated to assess the structural stability and flexibility. Figure 6a–c show that all of the docking complex's, TLR4's, and MEV's physical and chemical characteristics have reached equilibrium, meaning that their averages no longer vary with time. At the initial phase, RMSD of the docking complex was lower, and then, it began to rise to 20 ns before reaching a stable plateau from 50 ns (Fig. 6a). Meanwhile, because of the presence of adjuvants, the extremely flexible MEV construct has a significant RMSD (Fig. 6c). Conversely, the TLR4 receptor appears to be extremely stable right from the beginning of the MD simulation (Fig. 6b). Besides, the RMSF values for TLR4 and MEV were calculated at 0.143 ± 0.057 nm and 1.109 ± 0.518 nm, respectively, indicating higher flexibility in the vaccine than TLR4 (Fig. 6d). We also studied the compactness of the receptor TLR4 interaction with the MEV using Rg. The receptor remains compact, and no unusual folding or unfolding was observed throughout the 100 ns. Finally, the following formula was used to determine the buried surface area (BSA) at the TLR4-MEV interface, where the solvent accessible surface area (SASA) values were obtained by the GROMACS program:

$${\text{BSA}}_{{{\text{interface}}}} = \left( {{\text{SASA}}_{{{\text{TLR4}}}} + {\text{SASA}}_{{{\text{MEV}}}} } \right){-}{\text{SASA}}_{{{\text{TLR4}} - {\text{MEV}}}}$$

At the interaction interface between the MEV and TLR4, the BSA averaged 66.460 ± 8.982 nm^2^ in all three replicates of the simulation (Fig. 6f). Notably, Fig. 7 highlights the interface's enduring stability across the sampled snapshots, as demonstrated by the inter-residue interactions. The above analyses indicate the stability of interface interactions between the TLR4 and the vaccine construct. In summary, the MEV exhibited robust interactions and stability with the TLR4 receptor, as demonstrated by our triplicate MD simulations and molecular docking investigations.

## Conclusion

Leveraging the power of immunoinformatics, we have successfully designed a promising vaccine candidate that shows the potential to elicit a robust immune response against POWV. Through a comprehensive analysis of the POWV structural proteins, we identified key B-cell and T-cell epitopes. Then we employed these epitopes to construct a vaccine candidate that displayed remarkable antigenic potency while remaining non-toxic, non-allergenic, and possessing favorable physicochemical attributes. Molecular docking, molecular dynamics simulations, and immune simulations have provided evidence supporting the efficacy and safety of the formulated vaccine candidate. We hope this computational research provides a significant contribution to the field and that it will pave the way for in vitro and in vivo studies, ultimately leading to the development of a POWV vaccine that can be tested and potentially deployed in real-world scenarios, contribute to the global endeavor to combat and mitigate the emerging threat posed by POWV and arbovirus infections.

## Methods

### Retrieval and analysis of protein sequences

The polyprotein sequence of the Powassan virus was obtained from UniProt with the accession number Q04538. Since this research specifically focuses on the structural proteins, their sequence details were extracted from the PTM/Processing section within the UniProt entry. Additionally, the antigenicity and allergenicity of the structural proteins—namely, capsid, pre-membrane protein/membrane (prM/M), and envelope proteins—were evaluated using Vaxijen 2.0 and the allergenFP tool, respectively^37,38^.

### Prediction and evaluation of B-cell and T-cell epitopes

The NetMHC 2.3 website predicted T-helper cell epitopes that could bind to MHC class II molecules^39^. Additionally, the NetMHCpan 4.0 website was utilized to forecast T-cytotoxic cell epitopes that could potentially bind to MHC class I molecules^40^. The protein sequences were input in FASTA format, with selected peptide lengths of 9 and 15 (default), on the NetMHCpan 4.0 and NetMHC 2.3 web servers, respectively. The default parameters of the NetMHCpan 4.0 and NetMHC 2.3 web servers were applied to establish the thresholds for strong and weak binders. For the prediction of multiple linear B-cell epitopes, the IEDB B-cell epitope prediction website, employing the BepiPred 3.0 linear epitope prediction approach, was operated^41^. The Vaxijen version 2.0 web server was employed to determine the antigenicity of the B-cell and T-cell epitopes^37^. The allergenFP, ToxinPred, and IFNepitope web servers were used to evaluate the epitopes' allergic potential, toxicity, and interferon-γ activation potential, respectively^38,42,43^. The IL4Pred and IL-10Pred servers were utilized to assess the capability of the epitopes to induce interleukin-4 and interleukin-10 generation^44,45^.

### Formulation of the vaccine candidate and determination of the properties

The final epitopes, B-cell and T-cell, were joined to TpD adjuvant and *Escherichia coli* FimH protein with suitable linkers to formulate the final vaccine candidate. TpD is a universal adjuvant for TCD4 cells, possibly outperforming "PADRE", a peptide that binds to several HLA-DR (Human Leukocyte Antigen-DR) molecules promiscuously^46^. TpD has been shown in vitro to protect mucosal membranes, as well as to stimulate the generation of neutralizing antibodies^47^. These results are consistent in many mammalian species and are characterized by the development of durable CD4 + T memory cells, the production of neutralizing antibodies, and the release of cytokines such as TNF-α and interferon-gamma (IFN-γ). A T-helper cell (Th1)-dominant immunological response is indicated by this pattern^48^. To further enhance the immune stimulation of the vaccine, we included the final subunit of the type 1 fimbria of *Escherichia coli* (FimH), which has been shown to interact in a dependent way with TLR4. In comparison to lipopolysaccharide (LPS), this interaction regulates MHC class I and class II molecules more advantageously and securely and fosters the maturation, activation, and proliferation of peripheral and dendritic cells in the local area. Like TpD, FimH also promotes IFN-γ and TNF-α production^35,36^. Interestingly, FimH's application is effective in promoting mucosal immunity^36^. To form rigid or flexible protein configurations based on the desired biological activity, these adjuvants were linked by EAAAK linker; MHC-I and MHC-II epitopes were linked by KKK linker; and B-cell epitopes were linked by EAAAK linker as well. The physiochemical properties were analyzed by Expasy ProtParam webserver, whereas the antigenicity and allergic potential of the formulated vaccine candidate were determined by Vaxijen 2.0 and AllergenFP webservers^37,38,49^. The presence of a transmembrane helix in the vaccine candidate was predicted by DeepTMHMM webserver^50^. Furthermore, signal peptide in the vaccine construct was predicted by SignalP 4.1 tool^51^. Finally, BLASTp analysis is performed to identify the homologous protein of the vaccine candidate in humans (taxon id: 9606; *Homo sapiens*)^52^.

### In-silico immune simulation of the vaccine candidate

To assess the immune response induced by the formulated vaccine construct, we conducted an in-silico immune simulation using the C-ImmSim software^53^. We employed the default software settings except for the time step. Generally, it is recommended to maintain a minimum interval of four weeks between consecutive vaccine doses, although in certain situations, a longer gap may also be considered^54,55^. Thus, we evaluated the immune response profile for the vaccine construct by administering three vaccine doses at four-week intervals. Time steps of 1, 84 (approximately 4 weeks), and 168 (approximately 8 weeks) were utilized in the simulation.

### Modeling, refinement, and validation tertiary structure of the vaccine candidate

The 3D structure of the MEV construct was modeled using the AlphaFoldv2.0 tool^10,11^. Subsequently, to enhance the model's quality, the generated PDB file of the final vaccine from AlphaFold was submitted to the GalaxyRefine server for protein structure refinement^12^. This server initiates the reconstruction of all side chain structures, followed by iterative relaxation of the structure through brief molecular dynamics (MD) simulations after side chain repackage perturbations. Evaluation metrics provided by the GalaxyRefine server encompass global distance test-high accuracy (GDT-HA), root-mean-square deviation (RMSD), MolProbity (indicating crystallographic resolution), and Ramachandran favored score. RMSD measures the distance between atoms, with lower values indicating greater stability. An RMSD score falling from 0 to 1.2 is typically considered acceptable^12^. To supplement refinement, we use the ERRAT program to verify protein structures obtained through crystallography. It assesses errors based on non-bonded atom–atom interactions compared to a database of reliable high-resolution structures^56^. Additionally, the PROCHECK and ProSA-web servers were employed to assess the validity and quality of the selected 3D structure^57,58^. The Ramachandran plot analysis was conducted using the PROCHECK section of the UCLA-DOE LAB server. This plot illustrates the statistical distribution of backbone dihedral angles φ and ψ, along with the percentage and count of residues in the most favored, additionally allowed, generously allowed, and disallowed regions, thereby delineating the modeled structure's quality^57^. The ProSA-web server was also utilized to identify potential errors in the final vaccine structure^58^.

### Molecular docking and molecular dynamics simulation studies

For the docking of vaccine with TLR4, we utilized the PatchDock webserver, adhering to default settings^13^. It is a protein–protein docking algorithm and server that is used to predict the three-dimensional structure of protein complexes. The PatchDock algorithm employs a shape complementarity approach, where it matches complementary surface patches on the interacting proteins to generate potential docking poses^13^. By submitting two protein structures (vaccine and TLR4) to the PatchDock server, we can obtain predictions for their possible docking orientations and interaction interfaces. From this server, we got 10 models, which were ranked based on geometric shape complementarity and energy scoring.

Afterward, to evaluate the stability of the docked complex, molecular dynamics (MD) simulations were conducted using the GROMACS 2023 software on a Linux operating system^59^. Three replicas throughout 100 ns of the MD simulation were performed for all-atom to ensure the reliability of the data. The CHARMM27 all-atom force field was used to model the parameters of the proteins. There are 16,182 atoms and 1037 residues in total. The TLR4 protein complexed with the vaccine was placed in a cubic box 9 × 9 × 9 and solvated with SOL water molecules. The simulation box contained 380,145 atoms in the system. Charge neutralization was achieved by adding 2 Cl^-^ ions. System energy minimization was performed using the steepest descent method, applying a position restraint of 1000 kJ/mol nm^2^ on the heavy atoms of the protein. The equilibration process was carried out in a phased manner. Initially, a 1 ns NVT simulation was performed, followed by a 1 ns NPT simulation with restraints on the heavy atoms of the protein. Subsequently, a 10 ns equilibration without restraints on atoms was conducted using the NPT ensemble. The structure obtained after 10 ns of MD simulation was utilized as the starting structure for further equilibration and production simulations. Production simulations were run for 100 ns using the NPT ensemble, maintaining a temperature of 300 K with velocity rescaling and a coupling time of 0.1 ps. The pressure was maintained at 1 atm using a Parrinello-Rahman barostat with a coupling time of 2 ps. After the efficient completion of 100 ns MD simulations, calculations were performed for the RMSD of backbone residues, root mean square fluctuation (RMSF) of C-alpha, radius of gyration (Rg), and solvent accessible surface area (SASA). Besides, we conducted superimpositions of the complex structures extracted from selected snapshots during the simulation using GROMACS tools. Furthermore, to explore the interface connecting TLR4 and the vaccine construct, specific snapshots were analyzed using the COCOMAPS tool^60^.

**Figure 8:**
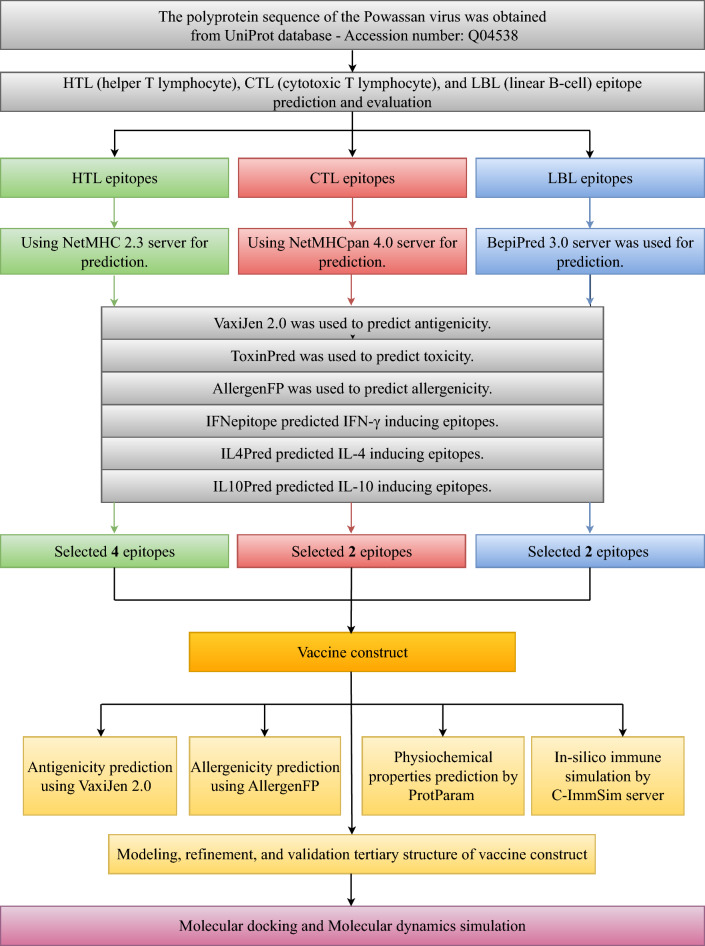
Schematic representation of the vaccine design workflow.

All methods used in this study are summarized in Fig. 8 below.

## Limitations

Designing an MEV via bioinformatics has some major disadvantages. Since all predictions are computationally based on methods involving mathematics, chemistry, and biology, the accuracy of in silico investigations never reaches 100%. Besides, as dynamic macromolecular complexes, proteins might have unexpected interactions in biological systems. The structure, function, and ability of proteins to associate with other proteins are all disrupted by changes in the physical environment, including pH, electrostatic charge, and the presence of different biomolecules, body fluids (such as blood and enzymes), which can occur in protein 3D conformations. Furthermore, antibodies are proteins that are specific to particular antigens, modifications to a single residue can prevent these antibodies from recognizing the antigen.

### Supplementary Information

Supplementary Information 1.

Supplementary Information 2.

## Data Availability

All data generated or analysed during this study are included in this published article (and its Supplementary Information files).
