# Supplementary material for: Immunoinformatics and computational approaches driven designing a novel vaccine candidate against Powassan virus
Source: Sci Rep. 2024 Mar 12;14:5999. doi: 10.1038/s41598-024-56554-9 (PMC10933373; doi:10.1038/s41598-024-56554-9)
Supplement: Supplementary file 1 — Supplementary Information 1. [file 41598_2024_56554_MOESM1_ESM.pdf]

Supplementary Information for

Immunoinformatics and computational approaches driven designing a novel vaccine candidate against Powassan virus

Truc Ly Nguyen[1], Heebal Kim[1,2,3*]

[1] Department of Agricultural Biotechnology and Research Institute of Agriculture and Life Sciences, Seoul National University, Seoul 08826, Republic of Korea

[2] Interdisciplinary Program in Bioinformatics, Seoul National University, Seoul 08826, Republic of Korea

[3] eGnome, Inc., Seoul, Republic of Korea

*Corresponding author:

- Postal address: Department of Agricultural Biotechnology, Seoul National University, Seoul 08826, Republic of Korea
- Telephone: +82 2 880 4802
- Fax: +82 2 883 8812
- E-mail address: heebal@snu.ac.kr
- ORCID: 0000-0003-3064-1303

I. Supplementary Figure

Figure S1. Predicted LDDT per residue for the 5 models of the vaccine’s 3D structure obtained from ColabFold v1.5.2: AlphaFold2.

Figure S2. Ramachandran plot for 3D structure of vaccine predicted by ColabFold v1.5.2: AlphaFold2.

II. Supplementary Table

Table S List of atom-atom interactions across the vaccine-TLR4 interface.

I. Supplementary Figure

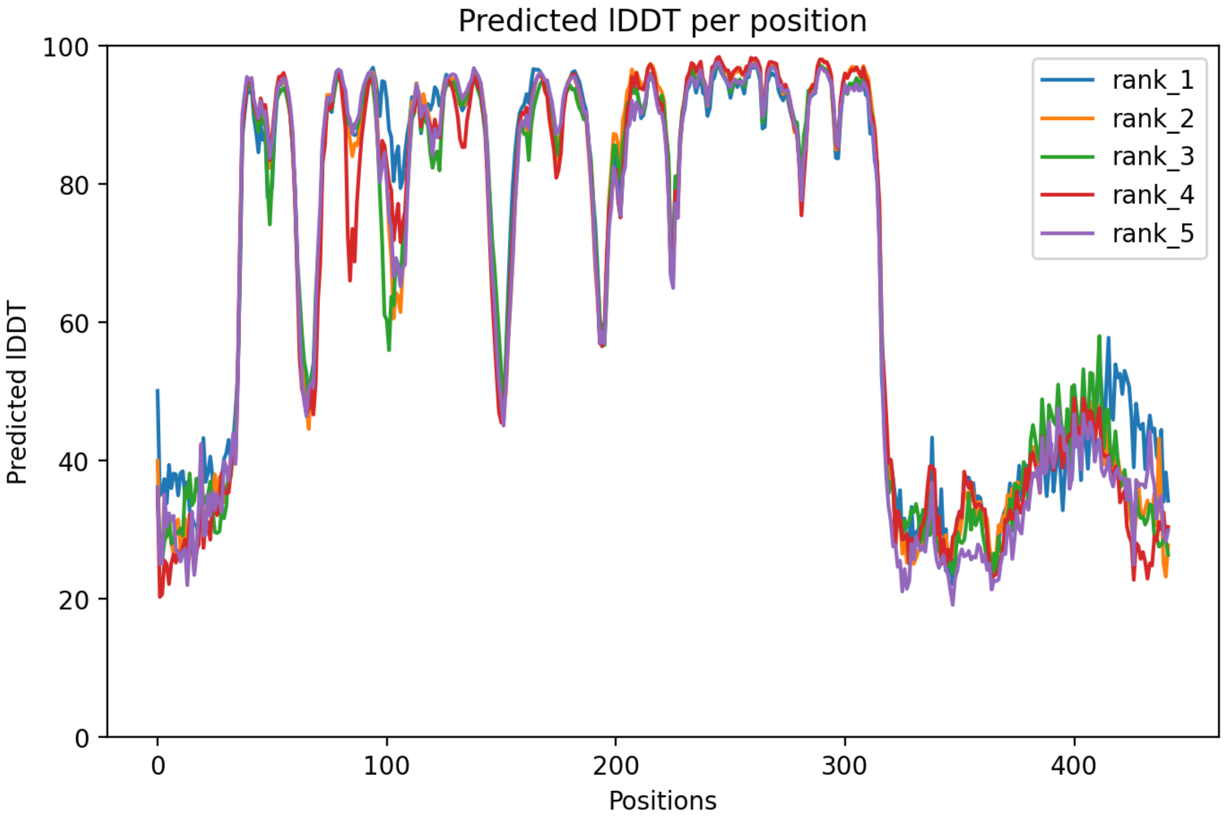

Figure S1. Predicted LDDT per residue for the 5 models of the vaccine's 3D structure obtained from ColabFold v1.5.2: AlphaFold2.

Ranking models by 'plddt' metric:

- rank_001_model_2_ pLDDT=69.6
- rank_002_model_3_ pLDDT=68.3
- rank_003_model_1_ pLDDT=68.1
- rank_004_model_5_ pLDDT=67.8
- rank_005_model_4_ pLDDT=67.4

PROCHECK

Ramachandran Plot

saves

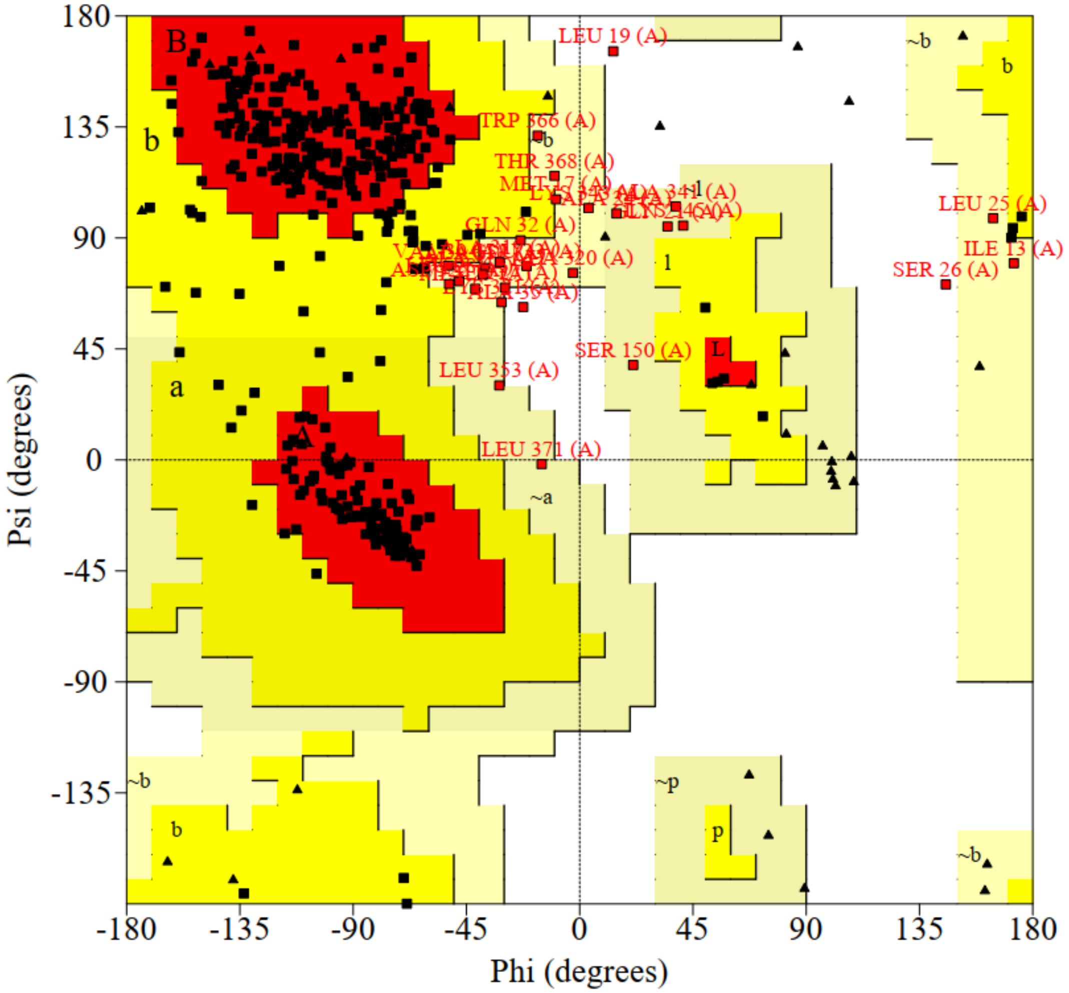

Plot statistics

Residues in most favoured regions [A,B,L]	301	79.8%
Residues in additional allowed regions [a,b,l,p]	48	12.7%
Residues in generously allowed regions [~a,~b,~l,~p]	24	6.4%
Residues in disallowed regions	4	1.1%
	----	------
Number of non-glycine and non-proline residues	377	100.0%
Number of end-residues (excl. Gly and Pro)	2	
Number of glycine residues (shown as triangles)	39	
Number of proline residues	24	

Total number of residues	442	

Based on an analysis of 118 structures of resolution of at least 2.0 Angstroms and R-factor no greater than 20%, a good quality model would be expected to have over 90% in the most favoured regions.

Figure S2. Ramachandran plot for 3D structure of vaccine predicted by ColabFold v1.5.2: AlphaFold2.

II. Supplementary Table

Table S List of atom-atom interactions across the vaccine-TLR4 interface.

**TLR4**					**Vaccine**				
**Hydrogen bonds**									
**No.**	**Atom no.**	**Atom name**	**Res. name**	**Res. no**	**Atom no.**	**Atom name**	**Res. name**	**Res. no**	**Distance**
1	6431	NH2	ARG	322	4	O	ILE	1	2.70
2	5985	N	ASN	268	260	O	MET	29	2.53
3	5111	ND1	HIS	159	366	NZ	LYS	41	2.30
4	5090	ND2	ASN	156	374	O	THR	42	3.30
5	5313	OG	SER	183	400	O	GLY	45	2.10
6	5299	OD2	ASP	181	402	N	THR	46	3.01
7	4890	NZ	LYS	130	493	O	TYR	58	2.57
8	4115	O	VAL	32	1004	NZ	LYS	113	2.63
9	8413	O	SER	569	2857	N	ALA	320	2.22
10	8645	NE2	GLN	597	2866	O	LYS	321	3.13
11	8609	NE1	TRP	593	2882	OD1	ASP	322	2.63
12	8380	NE2	GLN	565	2897	O	VAL	324	1.56
13	8570	OG	SER	589	2926	OE1	GLU	327	3.07
14	8355	OE1	GLN	562	2937	N	SER	329	2.65
15	7824	NH1	ARG	496	3639	O	LEU	396	2.90
16	7797	OG1	THR	493	3679	NZ	LYS	399	2.24
**Salt bridge**									
1	4575	NH2	ARG	87	1243	OD2	ASP	137	2.46
